# Genome annotation of *Aspergillus melleus* strain CBS 546.65

**DOI:** 10.1128/mra.00519-25

**Published:** 2025-08-25

**Authors:** Jason E. Stajich, Sean M. Garvey, Jéssica Gil-Serna

**Affiliations:** 1 Department of Microbiology and Plant Pathology, University of California 8790, Riverside, California, USA; 2 Institute for Integrative Genome Biology, University of California 117242, Riverside, California, USA; 3 Department of Research and Development, BIO-CAT, Inc. 53651 , Troy, Virginia, USA; 4 Department of Genetics, Physiology and Microbiology, Faculty of Biological Sciences, Complutense University of Madrid 111623 , Madrid, Spain; University of Strathclyde, Glasgow, United Kingdom

**Keywords:** *Aspergillus*, gene prediction, genome annotation

## Abstract

The fungus *Aspergillus melleus* is an important biosynthesis host for varied commercial applications. Gene annotation of a previously published genome produced 12,841 protein-coding genes and identified 102 biosynthetic gene clusters.

## ANNOUNCEMENT

**Fig 1:**
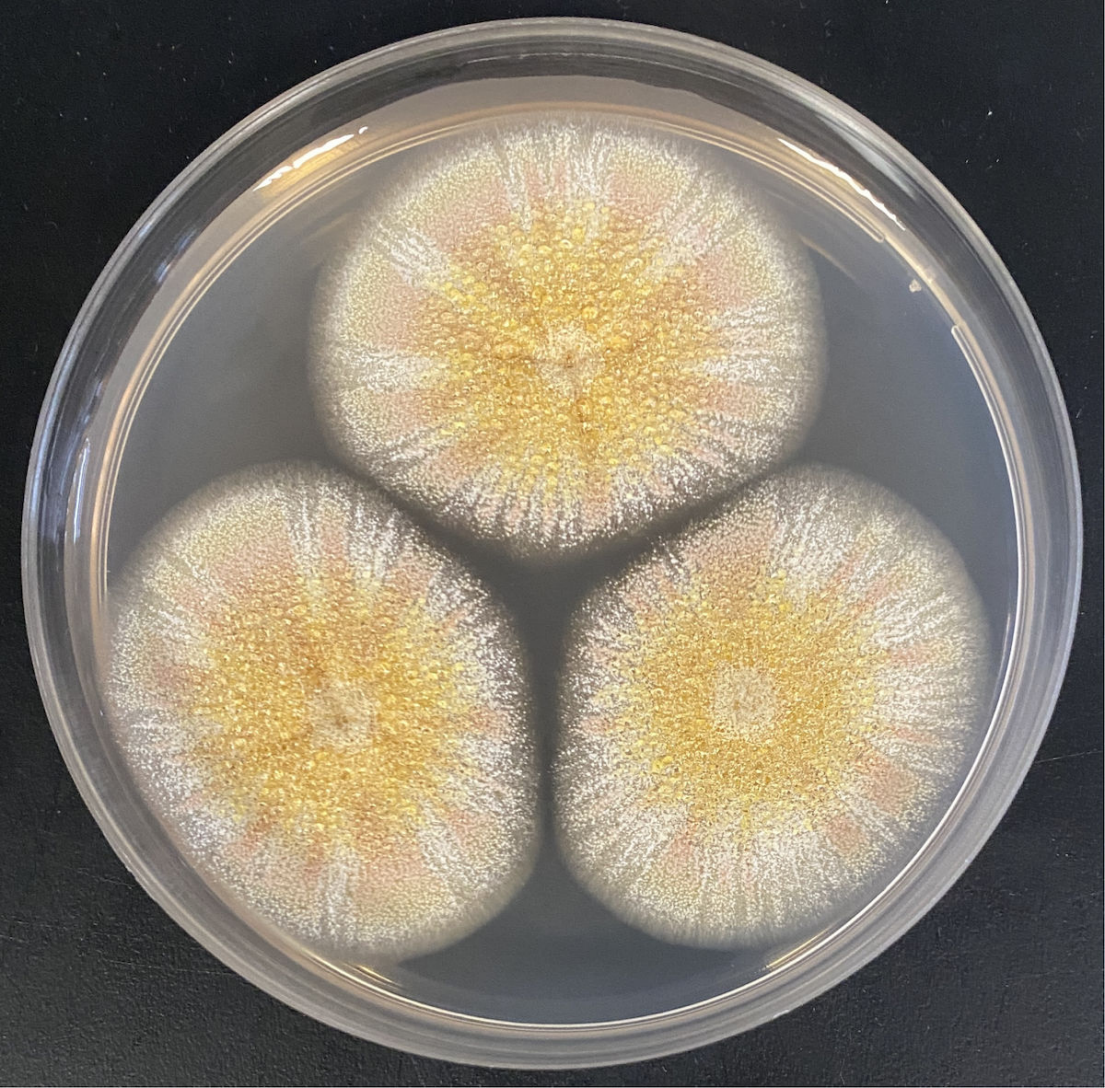
*Aspergillus melleus* CBS 546.65 colony after incubation for 7 days on potato dextrose agar.

*Aspergillus melleus* is an industrially important fungus that can synthesize the insecticide and nematicide compounds aspyrone (1) and mellamide (2), mycotoxins including ochratoxin A (3, 4), and antibiotic compounds (5). Secreted proteases obtained from *A. melleus* have also been developed as nutraceuticals to support immunity or digestive health (6–8). The neotype strain for the species was isolated from soil collected near Allahabad, India, in 1965 by B.S. Mehrotra and deposited by K.B. Raper and D.I. Fennell and deposited in the NRRL collection as NRRL 5103 (USDA-ARS Collection), American Type Culture Collection (ATCC) 16889, and CBS 546.65 in the Westerdijk Institute CBS collection (Fig. 1). The complete genome assembly was previously produced from a hybrid assembly of PacBio and Illumina DNA sequences but was not annotated (4).

Gene annotation was performed with Funannotate v1.8.8 (9) using a combination of RNA-seq and homologous protein sequences to inform gene prediction. The pipeline steps are archived in zenodo (10). First, the genome was masked for repetitive sequences and transposons by developing a *de novo* repeat library with RepeatModeler v2.0.1 (11) using default parameters and the “-LTRStruct” option. This library was used to lowercase mask repetitive genome regions by running RepeatMasker v4-1-1 (12) with Funannotate “mask.” Next, the Funannotate “train” step ran Trinity-GG v2.11.0 (13) and PASA v2.4.1 (14) to assemble transcripts from RNA-Seq alignments of *Aspergillus sesamicola*, a closely related species. Default Funannotate parameters were used to run Trimmomatic v0.39 (15), Hisat v2.2.1 (16), Kallisto v0.46.2 (17), Trinity-GG, and PASA to produce an assembly of 33,620 transcripts. The Funannotate “train” command trained gene prediction parameters for Augustus v3.3.3 (18), GlimmerHMM v3.0.4 (19), and SNAP v2013_11_29 (20) using the high-quality full-length PASA gene models. GeneMarkES-ET v4.62 (21) parameters were generated by its self-training protocol. Trinity-GG transcripts and Swissprot database protein sequences that matched the genome by diamond v2.0.13.151 BLASTX (22, 23) were aligned with the splice-site aware tool exonerate v 2.4.0 (24) and provided as exon evidence to *ab initio* gene predictors. CodingQuarry v2.0 (25) was run with the RNASeq alignment file from the “train” step. The gene predictions from all tools were combined to produce a composite set of 12,974 protein-coding genes by Evidence Modeler v1.1.1 (14). The predicted genes were filtered to remove proteins less than 50 amino acids in length or those with matches to transposases by diamond BLASTP of the Funannotate repeat library to produce a final set of 12,841 genes. The tool tRNAscan v1.3.1 (26) was run and predicted 213 non-overlapping tRNA gene models. The protein coding gene set was refined further with the Funannotate “update” step to add 5′- and 3′-untranslated region (UTR) exons to the gene models and predict alternatively spliced transcripts based on the Trinity transcripts and two rounds of PASA. The update step added 19 new gene models and 2,444 UTRs to produce a final data set of 13,061 genes and 13,395 transcripts (including the tRNA genes). Few genes had more than one isoform predicted (256), and there were 64 genes with at least three and 11 with four isoforms. To assign putative protein function, sequence similarity to UniProt v2021_02 (22), InterProScan v5.51-85.0 (27), EggNog v1.0.3 (28, 29), dbCAN v9.0 (30), and MEROPS v12.0 (37) databases was computed and associated with the protein sequences and CDS records. Prediction of biosynthetic gene clusters by antiSMASH v5.2.0 (31) identified 102 clusters, including 30 Type I polyketide synthases (PKS), 1 Type III PKS, 21 non-ribosomal peptide synthases (NRPS), 26 NRPS-like, 15 terpene, 6 indole, and 1 beta-lactone.

## Data Availability

This Whole Genome Shotgun project had been published and deposited previously at DDBJ/ENA/GenBank as accession JADPPX000000000. The record was updated from its initial deposit to include the gene predictions with permission of original authors. The RNA-Seq reads from *A. sesamicola* are associated with BioProject PRJNA585261 and SRA accessions SRR12142522 and SRR12142523. Annotation pipeline, antiSMASH results and logfiles are available in github https://github.com/stajichlab/Annotation_Aspergillus_melleus and archived at zenodo (10.5281/zenodo.15460055).
